# Food addiction in patients on weight loss treatment

**DOI:** 10.3389/fnut.2024.1459274

**Published:** 2024-10-16

**Authors:** Ana Cristina Palacio, Catalina Cuello, Ximena Díaz-Torrente

**Affiliations:** ^1^Carrera de Nutrición y Dietética, Facultad de Medicina, Universidad del Desarrollo, Santiago, Chile; ^2^Departamento de Cirugía Digestiva, Facultad de Medicina, Pontificia Universidad Católica de Chile, Santiago, Chile; ^3^Programa de Rehabilitación Cardíaca, Hospital Padre Hurtado—Facultad de Medicina, Universidad del Desarrollo, Santiago, Chile; ^4^Programa de Doctorado en Nutrición y Alimentos, Universidad de Chile, Santiago, Chile

**Keywords:** food addiction, YFAS 2.0, obesity, weight loss treatment, anthropometric measurements

## Abstract

**Background:**

Food addiction (FA) is defined as hedonic eating behavior involving the consumption of highly palatable foods (i.e., ultra processed foods in quantities beyond homeostatic energy requirements). FA is present in a subset of patients with overweight or obesity and could contribute to the response to weight loss treatments.

**Aim:**

Determine FA in individuals that fulfil the criteria of FA as measured by the YFAS 2.0, and its association with anthropometric and body composition variables in a clinical sample of patients undergoing weight loss treatment. Also, to determine the most prevalent FA criteria.

**Methods:**

An observational, analytical, and cross-sectional study was conducted. Sampling was non-probabilistic, convenience based. A total of 158 participants were recruited from two clinical centers (private and public) focused on managing non-communicable chronic diseases. The Chilean version of YFAS 2.0 was administrated through the online REDCap platform. Anthropometric measurements were measured, and body mass index (BMI) was calculated.

**Results:**

The mean age was 47.8 (SD 14.9) and BMI 28.7 (SD 5.3) kg/m^2^. There were 12.7% patients who screened positive on the YFAS 2.0 Chilean version (3.2% for mild, 2.5% for moderate and 7.0% for severe), the mean symptom count was 2.2 (SD 2.6), with *withdrawal* being the most prevalent criterion (33.5%). FA patients had significantly higher body weight compared to non-FA subjects (*p* = 0.045). We observed a slight and significant correlation between FA symptom count and body weight (*p* = 0.020), waist circumference (*p* = 0.005), and BMI (*p* = 0.023).

**Conclusion:**

This is the first study in Chile that showed that FA was present in patients undergoing weight loss treatment and was associated with anthropometric measurements. In addition, it showed that *withdrawal* was the most prevalent criterion. Future studies should investigate whether the presence of FA and the *withdrawal* criterion could contribute to suboptimal clinical response to weight loss treatment.

## 1 Introduction

Chile is one of the Latin American countries with the highest rates of chronic non-communicable diseases associated with a sedentary lifestyle and the consumption of foods and beverages with a high contribution of critical nutrients like salt/sodium, sugar, saturated fats and trans fats (i.e., UPF) (1). The greatest concern lies in the high rates of overweight and obesity (74% of the population over 15 years of age) reported by the latest National Health Survey, which represents a public health problem (2).

In recent years, evidence has emerged that UPF consumption—one of the main causes of overweight and obesity—could be explained, in part, by addiction to these UPF (3–7). The construct of Food Addiction (FA) refers to the fact that, in vulnerable individuals, i.e., those who, due to certain circumstances, personal or situational characteristics, are at greater risk of suffering harm. The consumption of these UPF activates an addictive response, similar to the response to abuse of substances, which leads to overconsumption (8, 9).

In the last decade, FA has become a growing focus of research due to its plausible relationship with the rise in obesity and other metabolic disorders (10, 11). The neurobiological determinants underlying this addiction include the activation and alteration of brain reward circuits, specifically the dopaminergic system. Studies have shown that UPF consumption can induce excessive dopamine release in the nucleus accumbens, a key region of the reward system (12). This dopamine release is similar to that observed in addictions to substances such as cocaine and nicotine, suggesting a common mechanism in the compulsive pursuit of external rewards (13). Moreover, continuous exposure to these foods can lead to decreased dopamine sensitivity, resulting in the need to consume larger quantities to achieve the same level of satisfaction, a phenomenon known as tolerance (14). This neuroadaptive process, combined with the activation of other neurochemical systems such as the opioid and endocannabinoid systems, contributes to the perpetuation of addictive behavior and impairs individuals’ ability to regulate their food consumption (15). Thus, for example, individuals with high body mass index (BMI) had the lowest D2 receptors values. Specifically, this reduction in striatal D2 receptors density correlates with reduced metabolism in cerebral areas (prefrontal and orbitofrontal cortex) that exert inhibitory control over consumption (16). Subjects with obesity show greater activation of reward and attention regions than normal-weight subjects do in response to hyper palatable food images versus control images (17). This observation suggests that a deficit in reward processing is an important risk factor for the addictive eating behaviors exhibited by individuals with obesity.

The conceptualization of FA has been based on theoretical models and assessment methods adapted from substance addiction. The Yale Food Addiction Scale (YFAS), developed by Gearhardt et al. (3), is a self-report scale designed to assess FA symptoms in the past 12 months, and has provided a validated tool to measure susceptibility to FA in the general population.

The most recent version, YFAS 2.0 (8), operationalizes the construct by applying the diagnostic criteria for substance use disorder from the Diagnostic and Statistical Manual of Mental Disorders (DSM-5) (9) (e.g., loss of control over consumption, intense cravings, withdrawal, continuing to use despite negative consequences). A recent meta-analysis estimated that 14% of adults in nonclinical samples met criteria for FA, with a higher prevalence (31%) in samples of adults with obesity (18).

Likewise, the literature shows a strong association between the FA symptom counts measured by YFAS 2.0 and obesity (1, 2). A systematic review that included clinical and non-clinical samples indicates that a higher BMI is associated with a greater symptom count of FA (19). In patients who are in the preoperative period of bariatric surgery (BS), the most frequent diagnostic criteria are the inability to control consumption, persistent desire, continuous consumption despite negative consequences and tolerance (20). Furthermore, FA symptoms have been reported to correlate positively and significantly not only with BMI, but also with body weight, waist and hip circumferences, body fat, and trunk fat percentages, suggesting that FA contributes to the severity of excess weight (21).

Modification of lifestyles, mainly diet and exercise, are recognized as the pillars of obesity treatment; however, a low percentage of individuals can permanently reduce excess body weight. Individuals who have followed a program to reduce their weight, 36 weeks after completing the intervention, regain the lost weight, recovering it completely after 1 year (22). On the other hand, it has been observed that individuals with FA have less success in traditional weight loss programs (20). Considering the assessment of FA construct becomes relevant in weight loss treatments, since meeting or not meeting the FA criteria may influence the short- and long-term weight loss outcome of the treatment. The integration of FA assessment into weight loss programs -may provide a more complete overview of the difficulties faced by patients, considering that the literature supports the association of FA with overweight and obesity. By understanding how FA influences eating behaviors, healthcare professionals can design more effective and personalized treatment strategies, thereby improving outcomes and treatment adherence. Thus, the aim of the present study was to evaluate FA in individuals that fulfil the criteria of FA as measured by the YFAS 2.0, and its association with anthropometric and body composition variables in a clinical sample of patients undergoing weight loss treatment. Considering that this is the first YFAS 2.0 report in a clinical sample of Chilean population, it seems appropriate to take into account which are the most prevalent criteria. In our view, this will allow us to configure a more detailed description of the FA condition. Our hypothesis was to find in this clinical sample a FA prevalence similar to that reported in other clinical samples and we expected to find a significant correlation between FA symptom count and the anthropometric measures. On this basis, this study aims to provide new knowledge from a psycho-nutritional perspective for people who are undergoing weight loss treatment due to their excess weight.

## 2 Methods

### 2.1 Design, participants and procedure

A cross-sectional study was conducted in a non-probabilistic convenience sample during the months of August to October 2022. The clinical sample was composed of patients undergoing weight loss treatment due to some health condition. They were recruited from two health care centers, a private one from a high socioeconomic sector and a public one from a medium-low socioeconomic sector. Participants were invited to participate when they were in the waiting room for a medical appointment corresponding to those included in the weight loss treatment program. They were told that this study was related to identifying different causes of overweight and obesity, and that participation was completely voluntary and without financial compensation; however, they would be given their anthropometric measurements. Those who were interested in participating were given informed consent. Participants who decided to participate, signed an informed consent. Once in the medical care room, they were asked to complete a questionnaire about demographic data; whether they were under psychological treatment; whether they had been diagnosed in the last 12 months with any of the following conditions: diabetes mellitus, insulin resistance, dyslipidemia, and/or hypertension; and whether they were taking any medication. After this questionnaire, they were given the Chilean version of YFAS 2.0 (23).

Once the scale was completed, anthropometric and body composition measurements were carried out by a nutritionist. A total of 158 individuals participated in this clinical sample (67.7% women; 15.2% diabetes mellitus; 20.3% insulin resistance; 32.9% hypertension; 25.3% hypercholesterolemia; 13.3% hypertriglyceridemia; 8.2% fatty liver; 8.2% polycystic ovary; 10.8% hypothyroidism; 84.8% taking medications).

The inclusion criteria were subjects over 18 years of age, who were in a weight loss treatment at the medical centers indicated above. Subjects who had any limitation, either in language or cognitive ability, that prevented self-administration of the questionnaire were excluded from the study. Additionally, pregnant, or lactating women were excluded.

### 2.2 Measurements

#### 2.2.1 Demographic data

Participants were asked to provide information on basic demographic data, including gender, age, marital status, educational level, and employment status.

#### 2.2.2 Anthropometric measurements and body composition

Weight and height were measured using a SECA^®^ brand scale (precision of 0.1 kg) and a stadiometer (precision of 0.1 cm). Nutritional status was determined by calculating the BMI. It was classified according to the World Health Organization criteria as underweight (BMI ≤ 18.5), normal nutritional status (BMI 18.5–24.9 kg/m^2^), overweight (BMI 25.0–29.9 kg/m2) or obesity (BMI ≥ 30 kg/m^2^) (24). Waist circumference (WC) was measured using a flexible tape, measured at the midpoint between the iliac crest and the last rib. The participants remained standing with their arms next to their body and their trunk free of clothing, and the measurement was carried out with their abdomen relaxed at the end of expiration (25).

To measure body composition, an InBody^®^ bioimpedance was used, determining the percentage of body fat mass and fat-free mass (i.e., muscle mass) (26). Nutritionists belonging to the weight loss treatment programs at each medical center performed the anthropometric and body composition measurements in the health care room, so that the privacy of the participants was protected during these measurements.

### 2.3 Chilean version of YFAS 2.0

The YFAS 2.0 is a 35-item self-report scale designed to assess FA symptoms over the previous 12 months based on the 11 diagnostic criteria for substance-related and addictive disorders proposed in DSM-5 and 1 clinical significance criterion (9). These FA criteria are *food consumed in larger quantities or over a longer period than intended*, *persistent desire or unsuccessful efforts to cut down or control consumption of certain foods*, *considerable time spent to obtain, consume, or recover from effects of food*, *giving up important social, occupational, or recreational activities because of food consumption*, *continuing to eat certain foods despite physical or psychological problems*, *tolerance*, *withdrawal*, *continued consumption despite social or interpersonal problems*, *failure to fulfill major role obligation*, *use in physically hazardous situations*, and *craving*, and *significant distress related to food*. This scale scored on an eight-level Likert scale (from 0 = never to 7 = every day). These scores produce two measurements: (a) a continuous symptom count score that reflects the number of diagnostic criteria met (ranging from 0 to 11); and (b) an FA threshold based on the number of symptoms (at least 2) and self-reported clinically significant impairment or distress. This final measurement allows for dichotomous classification of FA (FA vs. non-FA). Based on the DSM-5 taxonomy, the YFAS 2.0 also provides severity cutoffs for patients who exceed the FA threshold: mild (2 to 3 symptoms), moderate (4 to 5 symptoms), and severe (6 to 11 symptoms) (8). The Chilean version of the YFAS 2.0 has recently been validated, showing excellent psychometric properties (internal consistency KR20 0.85 and confirmatory factor analysis supported the unifactorial structure with fit indices of CFI = 0.975, TLI = 0.969, RMSEA = 0.056, with all factor loadings greater than 0.61) (23).

### 2.4 Statistical analysis

Stata version 16.0 (Stata Corp LLC, College Station, TX, United States) was used. The distribution of quantitative variables was evaluated using histograms and the Kolmogorov–Smirnov test. For statistical significance, a level of *p* < 0.05 was adopted.

The characteristics of the sample were represented in absolute values and percentages for qualitative variables and mean (SD: Standard Deviation) for continuous variables.

To detect differences between sociodemographic variables, anthropometric measurements, and body composition between subjects with and without FA, the Chi2, Fisher’s Exact, Student’s T or ANOVA test was used, depending on the type of variable. Spearman correlations between FA symptom counts and anthropometric and body composition measurements were assessed. As sensitivity analysis, we considered correlations without individuals with normal weight.

## 3 Results

Table 1 shows demographic and anthropometric characteristics of the sample. The largest proportion of participants were women (67.7%), employed (68.4%), married (56.3%) and with an educational level of completed university studies (51.2%). The mean age was 47.8 (SD 14.9) and BMI 28.7 (SD 5.3) kg/m^2^.

**Table 1:** Sociodemographic characteristics and anthropometric measurements in clinical sample (*n* = 158).

Variables		
Gender, % (*n*)	Female	67.7 (107)
	Male	32.3 (51)
Age, (M ± SD)	Years	47.8 (14.9)
Educational level, % (*n*)	Primary or less	14.6 (23)
	Secondary	34.2 (54)
	University	51.2 (81)
Employment situation, % (*n*)	Unemployed	29.7 (47)
	Employed	68.4 (108)
	University Students	1.9 (3)
Marital status, % (*n*)	Single	32.3 (51)
	Married	56.3 (89)
	Divorced	8.9 (14)
	Widower	2.5 (4)
Psychological treatment, % (*n*)	No	63.9 (101)
	Yes	36.1 (57)
Weight status, % (*n*)	Normal weight	20.9 (33)
	Overweight	37.3 (59)
	Obesity	41.8 (66)
Anthropometric measures, (M ± SD)	Weight (kg)	75.5 (15.2)
	BMI (kg/m2)	28.7 (5.3)
	WC (cm)	93.9 (10.8)
	Body fat (%)	36.2 (7.9)
	Lean mass (kg)	26.3 (5.7)

Table 2 shows 12.7% FA prevalence and means symptom count 2.2 (SD 2.6). Regarding the severity of FA, the percentages were distributed as 3.2% for mild, 2.5% for moderate and 7.0% for severe. Regarding the number of symptoms reported, the most frequently reported were: 33.5% *withdrawa*l, 31.0% *persistent desire or unsuccessful efforts to reduce or control the consumption of certain foods*, 27.9% *continuous consumption despite social or interpersonal problems*.

**Table 2:** Prevalence and symptom count of food addiction in clinical sample of adults (*n* = 158) according to the Chilean YFAS 2.0 scale.

Characteristics	% (*n*)
Prevalence FA	12.7 (20)
Mild	3.2 (5)
Moderate	2.5 (4)
Severe	7.0 (11)
Symptom count	2.2 (2.6)
Criteria	
Food consumed in larger quantities or over a longer period than intended	26.6 (42)
Persistent desire or unsuccessful efforts to cut down or control consumption of certain foods	31.0 (49)
Considerable time spent to obtain, consume, or recover from effects of food	22.2 (35)
Giving up important social, occupational, or recreational activities because of food consumption	10.1 (16)
Continuing to eat certain foods despite physical or psychological problems	21.5 (34)
Tolerance	15.2 (24)
Withdrawal	33.5 (53)
Continued consumption despite social or interpersonal problems	27.9 (44)
Failure to fulfill major role obligation	7.6 (12)
Use in physically hazardous situations	17.1 (27)
Craving	10.8 (17)
Significant distress in relation to food	16.5 (26)

Table 3 shows the demographic and anthropometric characteristics according to fulfilment of criteria of FA (FA) or not fulfilment (non-FA). The patients with FA were significantly younger than the non-FA subjects (*p* = 0.025). Furthermore, FA patients had significantly higher body weight compared to non-FA subjects (*p* = 0.045).

**Table 3:** Sociodemographic characteristics and anthropometric measurements according to no food addiction or food addiction in clinical sample (*n* = 158).

Variables		No food addiction *n* = 138	Food addiction *n* = 20	*p* value^a^
Gender, % (*n*)	Female	66.7 (92)	75.0 (15)	0.456
	Male	33.3 (46)	25.0 (5)	
Age, (M ± SD)	Years	48.8 ± 15.2	40.9 (10.7)	**0.025**
Educational level, % (*n*)	Primary or less	15.2 (21)	2.0 (10)	0.514
	Secondary	35.5 (49)	5.0 (25)	
	University	49.3 (68)	13.0 (65)	
Employment situation, % (*n*)	Unemployed	31.9 (44)	3.0 (15)	0.140
	Employed	66.7 (92)	16.0 (80)	
	University Students	1.4 (2)	1.0 (5)	
Marital status, % (*n*)	Single	31.9 (44)	35.0 (7)	0.650
	Married	57.2 (79)	50.0 (10)	
	Divorced	8.0 (11)	15.0 (3)	
	Widower	2.9 (4)	0.0 (0)	
Psychological treatment, % (*n*)	No	65.2 (90)	55.0 (11)	0.374
	Yes	34.8 (48)	45.0 (9)	
Weight Status, % (*n*)	Normal weight	21.7 (30)	15.0 (3)	0.678
	Overweight	37.7 (52)	35.0 (7)	
	Obesity	40.6 (56)	50.0 (10)	
Anthropometric measures, (M ± SD)	Weight (kg),	74.6 (14.0)	82.5 (21.4)	**0.045**
	BMI (kg/m2)	28.5 (4.6)	30.7 (9.0)	0.114
	WC (cm)	93.4 (10.4)	97.8 (12.8)	0.164
	Body fat (%)	35.9 (8.0)	38.6 (7.2)	0.290
	Lean mass (kg)	25.9 (5.5)	29.1 (7.1)	0.082

^aChi-square, Fisher Exact or T-Student test depending on the type of variable for the difference of sample; BMI: Body Mass Index. WC: Waist Circumference. In bold type, p < 0.05.^

The symptom count according to demographic and anthropometric characteristics is presented in Table 4, showing no significant differences between FA and Non-FA subjects.

**Table 4:** Symptom count of food addiction according to demographics characteristics and anthropometric measurements in clinical sample (*n* = 158).

Variables		Symptom count (M ± SD)	*p* value
Gender	Female	2.47 (2.67)	0.086
	Male	1.72 (2.30)	
Educational level	Primary or less	2.13 (2.60)	0.056
	Secondary	1.67 (2.06)	
	University	2.64 (2.82)	
Employment situation	Unemployed	1.83 (2.18)	0.110
	Employed	2.38 (2.74)	
	University students	3.33 (1.15)	
Marital status	Single	1.96 (2.41)	0.151
	Married	2.31 (2.61)	
	Divorced	3.07 (3.08)	
	Widower	1.00 (0.81)	
Psychological treatment	No	2.13 (2.41)	0.752
	Yes	2.42 (2.84)	
Weight status	Normal weight	1.79 (2.47)	0.219
	Overweight	1.98 (2.27)	
	Obesity	2.68 (2.83)	

T-student test or ANOVA test.

**Figure 1:**
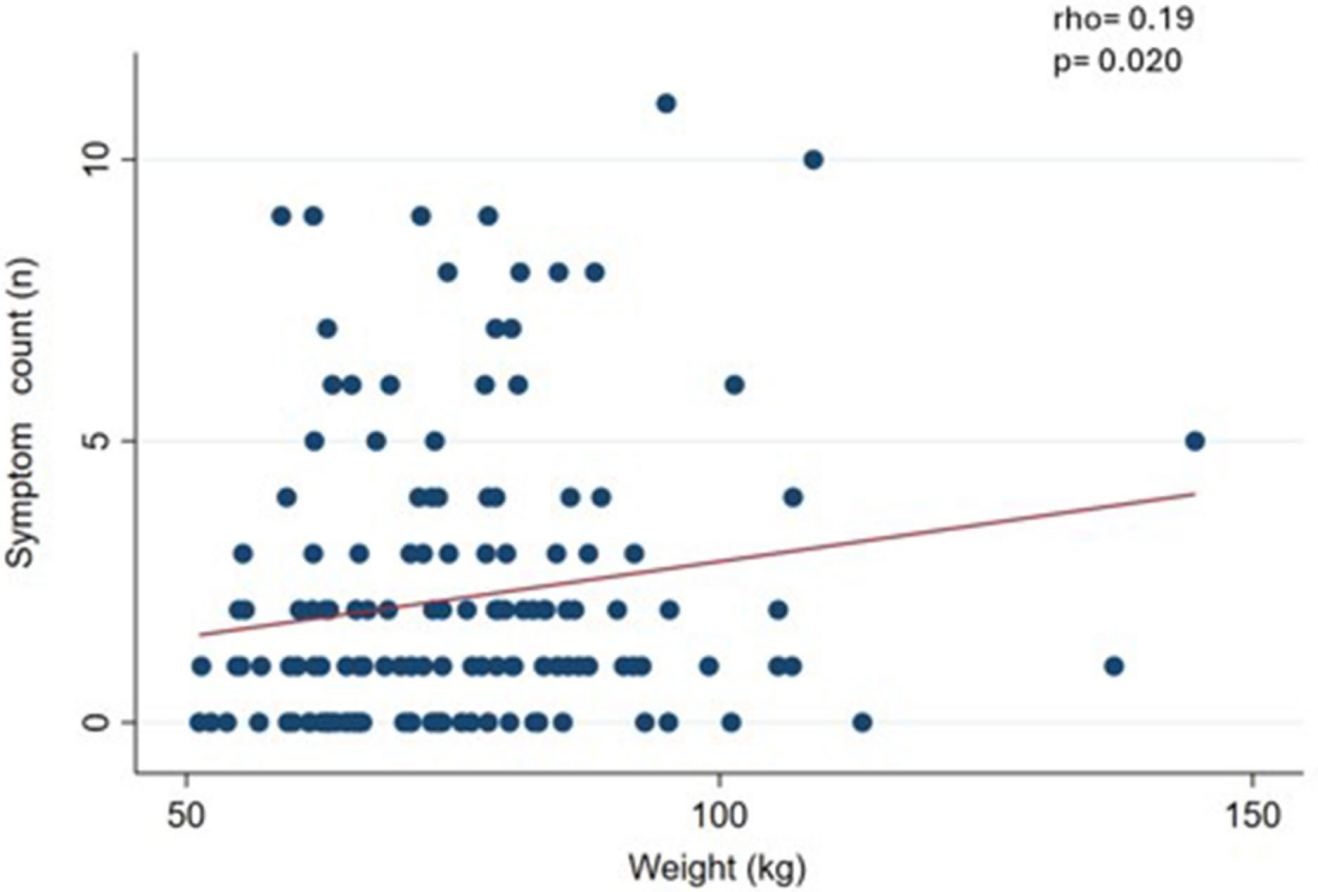
Correlation between food addiction symptom count and weight in clinical sample (*n* = 146).

**Figure 2:**
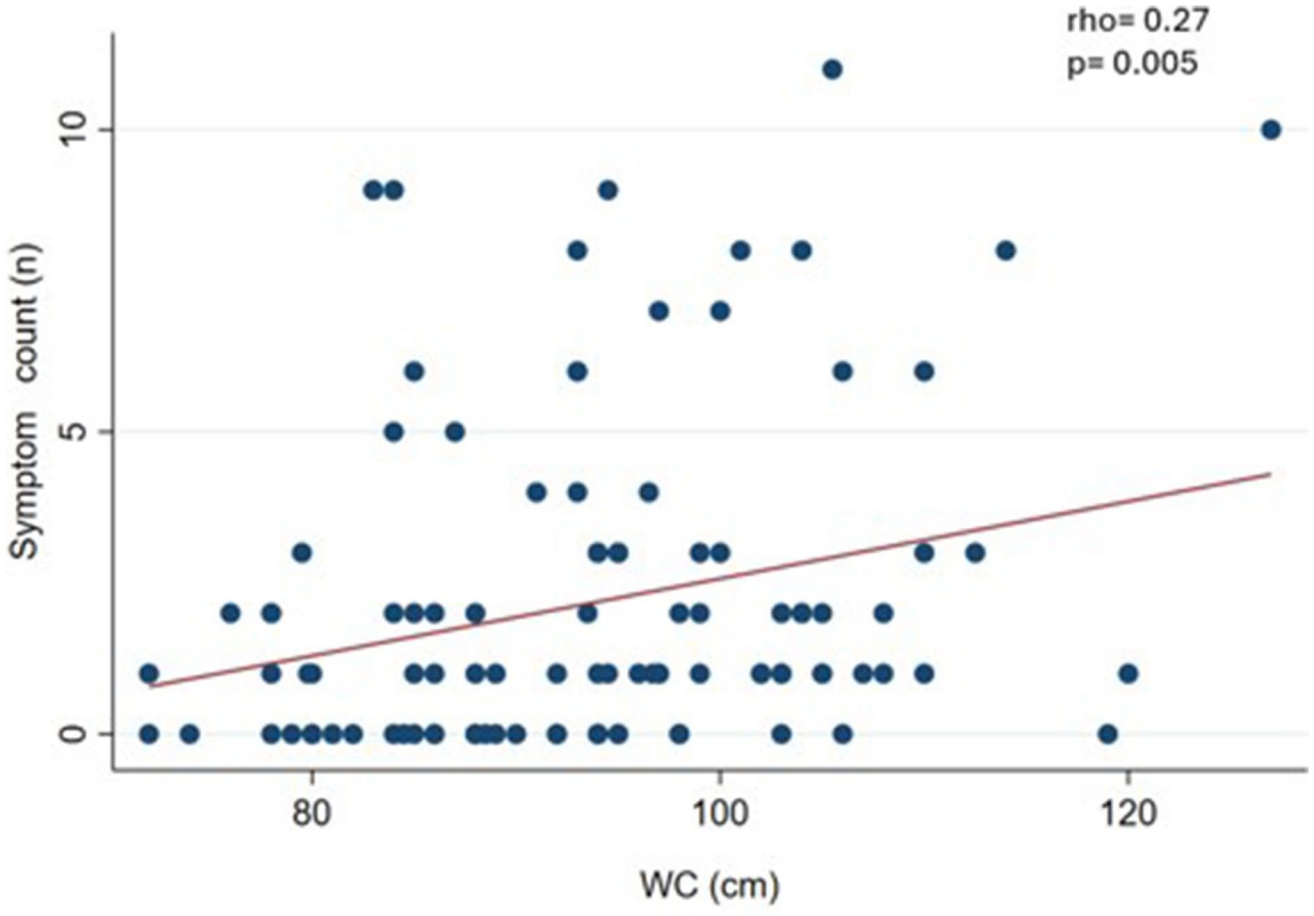
Correlation between food addiction symptom count and waist circumference (WC) in clinical sample (*n* = 108).

**Figure 3:**
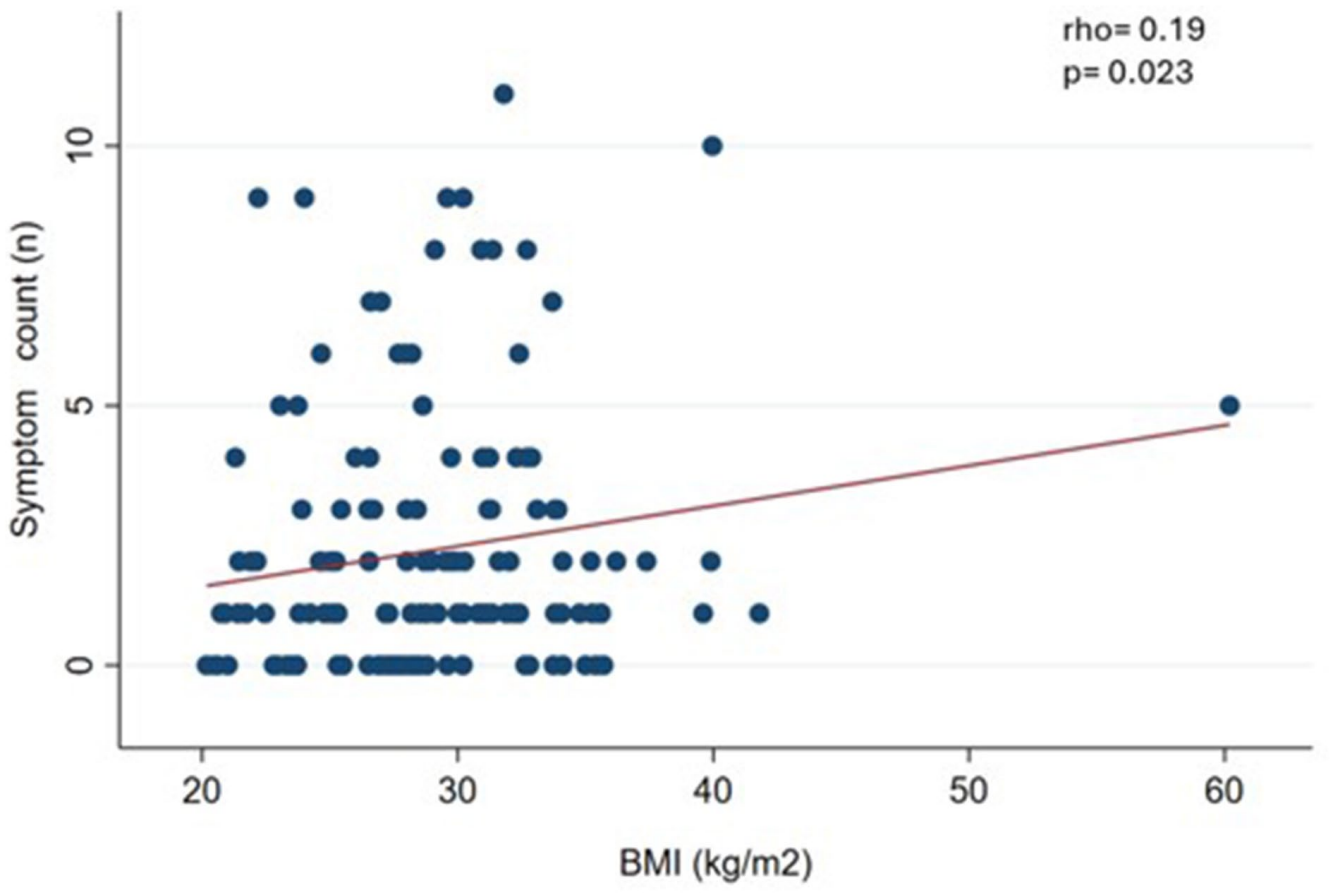
Correlation between food addiction symptom count and body mass index (BMI) in clinical sample (*n* = 144).

We observed a slight and significant correlation (*p* < 0.05) between symptom count and body weight (rho = 0.19) (Figure 1), waist circumference (rho = 0.27) (Figure 2), and BMI (rho = 0.19) (Figure 3).

As a sensitivity analysis, we performed the correlations without considering normal-weight subjects. The result was that WC maintained a significant correlation (rho = 0.30; *p* = 0.006) (data not shown in figures).

## 4 Discussion

In this clinical sample of patients undergoing treatment for body weight loss, the prevalence of FA was 12.7%, with severe diagnosis being predominant severity. The most predominant diagnostic criterion was *withdrawal*. Furthermore, we found a relationship between FA symptom count, body weight, BMI, and WC. These results partially confirm our hypothesis, since the prevalence of FA was somewhat lower than expected and there was correlation between the FA symptom count and some anthropometric measures.

Depending on whether the samples are clinical or non-clinical, the literature reports FA prevalence of 31% vs. 14%, respectively (16). A recent meta-analysis that included Latin American countries shows FA prevalences of 5.9 to 95.3% in clinical samples (27). This wide range of prevalence can be attributed to the diverse characteristics of the included participants, since some were in treatments related to cardiovascular health (28), binge eating disorders (29) or evaluation for BS (30, 31). In our study, patients were undergoing treatment to reduce their body weight, for reasons of cardiovascular health or for management of metabolic syndrome. This could explain the lower prevalence compared to those reported for other clinical samples, mainly those that include candidates to BS (30, 31). Among the participants who met the diagnostic criteria for FA, 7% of them were found in the severe category, similar to what is reported in the literature (32).

In the present study 2.2 (SD 2.6) symptom count were observed, lower than that reported by Pursey et al. in a meta-analysis in clinical samples a mean of 4.0 (SD 0.5) symptoms (33). This difference may be attributed to the lower BMI of our sample.

The FA diagnostic criterion that predominated in this clinical sample was *withdrawal* (33.5%), which refers to aversive physical, cognitive, and affective symptoms that arise after reduction or discontinuation of an addictive substance. This symptom was one of the most common in the original validation study (29.7%) and in a sample of the general Italian population (12.5%) (34). *Withdrawal* has great clinical relevance as a predictor of relapse and is a target for intervention in substance use disorders (35, 36). In the context of hyperpalatable foods, *withdrawal* has received less research than has been done with addictive drugs and may be an important area for future research with clinical relevance. The second criterion that occurred in the highest proportion (31.0%) was *persistent desire or unsuccessful efforts to reduce or control the consumption of certain foods*. This symptom has been reported in 91.7% in a sample of university students (7), and in 25.0% of the original validation study sample (8). Like consuming more than planned or losing control, this symptom frequently occurs in subjects with eating-related problems and could be triggered by the availability of hyperpalatable foods (37). The third criterion that predominated (27.9%) was *continued consumption despite social or interpersonal problems*. This shows the complexity of addiction, even when individuals are aware of how it can negatively impact their lives.

Our results showed no differences in the prevalence of FA between women and men. Scientific evidence shows diverse results depending on sex, some indicating that FA is more prevalent in women (8, 38, 39), while others have found no differences by sex (40). In the present study, the mean age of those who presented FA was 40.9 vs. 48.8 of those who did not present FA. The literature indicates that FA is more prevalent at younger ages (3).

Regarding nutritional status, no differences were found according to FA or non-FA. BMI also did not differ by FA diagnosis. The literature presents mixed results; some cross-sectional studies have reported that the probability of FA in participants with obesity is greater than in subjects with normal weight (40–42). However, other studies do not observe these differences according to nutritional status (43, 44). These mixed results could suggest that FA could be associated with other binge eating patterns beyond body weight. Minhas et al. indicate that FA appears to be associated with elevations in impulsivity, particularly deficits in emotional regulation (45).

Furthermore, the symptom count of FA was correlated with weight, BMI, and WC measurements. Cross-sectional design studies show a positive and significant correlation between symptom count of FA and BMI (31, 46, 47). It seems incongruent that in this sample the symptom count of FA was associated with weight-related measures (such as BMI and WC), while FA (as a dichotomous measure) does not show differences by nutritional status. Well, the literature indicates that the symptom count of FA seems to be more sensitive to detect these associations, particularly in samples of participants where the prevalence of FA is not very high (between 10 and 15%) (46, 48, 49).

On the other hand, we performed a sensitivity analysis eliminating subjects with normal weight from the statistical analyses. Correlations between FA symptom count, weight and BMI lose statistical significance, whereas WC maintains significance. This demonstrates that WC assessment ends up being relevant, given that it proved to be a robust indicator associated with FA. WC measurement could be a valuable tool for assessing FA due to its ease of collection and its relevance in the identification of cardiovascular risk. Although few studies have explored this relationship (45), our investigation has found a significant association between both variables. WC, as an indicator of metabolic health, not only reflects the physical state of the individual, but could also be a useful marker to identify eating behavior patterns. Therefore, we propose that its inclusion in clinical assessment could provide a comprehensive perspective in the management of cardiovascular risk and in FA treatment.

Besides, the study of FA may underlie behaviors that lead to cardiovascular risk factors, and is a possible psychological factor associated with cardiovascular diseases. Evidence suggests that individuals with FA often engage in overeating and poor dietary choices, contributing to obesity, hypertension, and dyslipidemia, all of which are established risk factors for cardiovascular diseases (7). Additionally, the compulsive eating behaviors seen in FA are linked to increased inflammatory markers, which play a crucial role in the development and progression of atherosclerosis. A recent review identified a positive relationship between C-reactive protein (CRP)/high-sensitivity CRP and loss of control eating which is one of the criteria for FA. Other inflammatory markers that potentially have a positive relationship with obesity-related eating behaviors include fractalkine and fibrinogen (50). On the other hand, research has shown a significant association between FA and increased risk of metabolic syndrome, a cluster of conditions that elevate the risk of heart disease, stroke, and diabetes (21). Therefore, understanding FA and its impact on cardiovascular health is essential for developing comprehensive strategies to mitigate these risks.

The search for the ideal treatment for weight loss and its long-term maintenance have been the aim of numerous investigations. New causes and consequences of overweight and obesity are frequently revealed that must be treated to achieve success in this type of treatment. This is why the FA’s role as a contributor must be investigated and addressed (42), considering that the diagnosis of FA could affect adherence to treatment and therefore its effectiveness in people seeking to lose weight (51).

Gearhardt and Hebebrand have conducted an interesting debate as to whether FA is a distinct construct. In this debate, data are provided on the activation of the reward system in the brain in response to certain foods (i.e., UPF), as well as the presence of addictive behaviors such as loss of control and compulsive seeking of certain foods, but disagreement remains as to the strength of the evidence that UPF are addictive (52). While we support the idea that more evidence is needed regarding that UPF may have addictive effects, we believe that in a subset of the population these effects are significant enough to justify inclusion of the FA construct in obesity treatment.

Early recognition and selection of patients with greater barriers to controlling body weight is important to design strategies that contribute to improving treatment outcomes (53). On the other hand, it would be important to determine if FA predicts the probability of abandoning weight control programs, considering that the dropout rate of subjects who are in weight loss programs is generally high (30%) (53).

Our results must be interpreted considering certain limitations, typical of cross-sectional studies, which do not allow us to draw causal inferences. First, we do not have information regarding how long the subjects had been in the weight loss program, which could explain, in part, the presence of normal weight subjects in this clinical sample. Furthermore, no sample size calculation was performed due to time and cost considerations. Thus, our sample size may not have sufficient power to determine other potential expected associations, for example, the association between body fat percentage and FA.

As a strength we can mention that, to our knowledge, this is the first study to evaluate FA using YFAS 2.0 Chilean version in a clinical sample. This instrument has undergone rigorous psychometric testing and has strong internal consistency and inter-test reliability, as well as convergent, discriminant, and incremental validity (23). It has been translated into more than 12 languages, such as Spanish, Persian and Chinese, and these versions also show strong psychometric properties (54–56).

## 5 Conclusion

This is the first study in Chile that assessed in a clinical sample the association between FA and different anthropometric measurements, also showing the most prevalent FA criteria. We observed the presence of FA diagnostic criteria in this sample of patients undergoing weight loss treatment, with “*withdrawal”* being the most prevalent criterion. Moreover, FA symptom count was associated with key anthropometric measures such as weight, WC, and BMI. These findings highlight the clinical relevance of FA in individuals on a weight loss program, underscoring the need for tailored multidisciplinary interventions. It also highlights that “*withdrawal*” should be addressed in future studies. Addressing FA in this population may improve treatment outcomes, allowing for more personalized therapeutic strategies that take into account the presence of addictive eating behaviors. This approach could optimize resource allocation and facilitate the development of timely and effective interventions that support long-term weight control and improved health, aligning with the growing evidence linking FA to overweight and obesity.

## Data Availability

The raw data supporting the conclusions of this article will be made available by the authors, without undue reservation.
